# Supplementary material for: Understanding COVID-19 vaccination behaviors and intentions in Ghana: A Behavioral Insights (BI) study
Source: PLoS One. 2024 Feb 9;19(2):e0292532. doi: 10.1371/journal.pone.0292532 (PMC10857727; doi:10.1371/journal.pone.0292532)
Supplement: S1 Appendix — (DOCX) [file pone.0292532.s002.docx]

**Appendix A. Correlations between measures of drivers for participants who reported being vaccinated**

|  | **M** | **SD** | **1.** | **2.** | **3.** | **4.** | **5.** | **6.** | **7.** | **8.** |
| --- | --- | --- | --- | --- | --- | --- | --- | --- | --- | --- |
| 1. Risk perception | **3.75** | **1.39** |  |  |  |  |  |  |  |  |
| 2. Effectiveness | **3.8** | **1.26** | **0.64** |  |  |  |  |  |  |  |
| 3. Ease of Vaccination | **3.66** | **1.35** | **0.69** | **0.58** |  |  |  |  |  |  |
| 4. Own attitudes | **4.17** | **1.26** | **0.71** | **0.70** | **0.70** |  |  |  |  |  |
| 5. Family’s attitudes | **4.10** | **1.27** | **0.70** | **0.66** | **0.69** | **0.87** |  |  |  |  |
| 6. Community’s attitudes | **4.04** | **1.27** | **0.67** | **0.64** | **0.67** | **0.85** | **0.86** |  |  |  |
| 7. Religious leaders’ attitudes | **4.14** | **1.26** | **0.70** | **0.67** | **0.70** | **0.87** | **0.85** | **0.87** |  |  |
| 8. Healthcare providers’ attitudes | **4.21** | **1.21** | **0.68** | **0.65** | **0.67** | **0.86** | **0.84** | **0.83** | **0.88** |  |

*Note. N=1089*

**Appendix B. Correlations between measures of drivers for participants who reported being unvaccinated.**

|  | **M** | **SD** | **1.** | **2.** | **3.** | **4.** | **5.** | **6.** | **7.** | **8.** |
| --- | --- | --- | --- | --- | --- | --- | --- | --- | --- | --- |
| **1.Risk perception** | **3.20** | **1.32** |  |  |  |  |  |  |  |  |
| **2.Effectiveness** | **3.21** | **1.34** | **0.63** |  |  |  |  |  |  |  |
| **3.Ease of Vaccination** | **3.09** | **1.32** | **0.69** | **0.61** |  |  |  |  |  |  |
| **4.Own attitudes** | **3.38** | **1.40** | **0.68** | **0.74** | **0.74** |  |  |  |  |  |
| **5.Family’s attitudes** | **3.30** | **1.39** | **0.70** | **0.72** | **0.73** | **0.88** |  |  |  |  |
| **6.Community’s attitudes** | **3.26** | **1.40** | **0.73** | **0.69** | **0.75** | **0.85** | **0.90** |  |  |  |
| **7.Religious leaders’ attitudes** | **3.38** | **1.38** | **0.66** | **0.68** | **0.69** | **0.85** | **0.84** | **0.83** |  |  |
| **8.Healthcare providers’ attitudes** | **3.63** | **1.30** | **0.65** | **0.68** | **0.64** | **0.80** | **0.78** | **0.76** | **0.83** |  |

*Note. N=405*

**Appendix C**

Below are the BI message types that survey participants were randomly assigned to. All BI message types were presented in the language selected by the participant at the beginning of the survey.

##### Fear

- “Without the vaccine you put your health at risk for more severe forms of COVID-19 and death. Get Vaccinated!”

1. Altruism

- “By taking the vaccine, you protect your loved ones from the more severe forms of COVID-19 and death.Get Vaccinated!.”

1. Social Norms

- “The majority of people in your community are supporting or planning to get the vaccine as soon as possible.”

1. Messenger Effect: Ghana Health Service

- “Vaccination is strongly recommended by the Ghana health service as an effective tool to protect yourself from severe forms of COVID-19. Get Vaccinated!”

1. Messenger Effect: Doctor, First-person

- “Hi! I’m a doctor, coming to you today with an important message. Doctors all across Ghana recommend vaccination as an effective tool to protect yourself from severe forms of COVID-19. Get Vaccinated!”

1. Messenger Effect: Religious Leaders

- "Vaccination is strongly recommended by priests, pastors, and imams as an effective tool to protect yourself from severe forms of COVID-19. Get Vaccinated!

**Appendix D. Differences in the Relative Importance of each Driver of Vaccination Behavior**

| ***Drivers*** | ***Raw Importance*** | ***Rescaled Importance*** | ***Risk Perception*** | ***Effectiveness*** | ***Ease of Vaccination*** | ***Own attitudes*** | ***Family’s attitudes*** | ***Community’s attitudes*** | ***Religious leaders’ attitudes*** | ***Healthcare providers’ attitudes*** |
| --- | --- | --- | --- | --- | --- | --- | --- | --- | --- | --- |
|  |  |  | *Difference* | *Difference* | *Difference* | *Difference* | *Difference* | *Difference* | *Difference* | *Difference* |
| 1.Risk perception | .005 | 5.74% |  | -.002 | -.0003 | -.010* | -.011* | -.008* | -.009* | -.004* |
| 2.Effectiveness | .008 | 9.67% |  |  | .002 | -.008* | -.009* | -.007 | -.007 | -.003 |
| 3.Ease of Vaccination | .006 | 6.86% |  |  |  | -.010* | -.011* | -.008* | -.009* | -.004* |
| 4.Own attitudes | .014 | 17.48% |  |  |  |  | -.001 | .002 | .001 | .006 |
| 5.Family’s attitudes | .015 | 18.56% |  |  |  |  |  | .003 | .002 | .007 |
| 6.Community’s attitudes | .014 | 16.48% |  |  |  |  |  |  | -.001 | .004 |
| 7.Religious leaders’ attitudes | .014 | 16.66% |  |  |  |  |  |  |  | .005 |
| 8.Healthcare providers’ attitudes | .007 | 8.55% |  |  |  |  |  |  |  |  |

*Note. (N* = 1494), Difference. is computed by subtracting the raw importance of one predictor from the other.

*Indicates that the confidence interval for difference does not include 0, indicating that the difference of raw importance between the two predictors is significant.

**Appendix E. Results of the Relative Weights Analysis (RWA) and confidence intervals: Vaccination Intention**

| ***Drivers*** | ***Raw Importance*** | ***Rescaled Importance*** | ***Risk perception*** | ***Effectiveness*** | ***Ease of Vaccination*** | ***Own attitudes*** | ***Family’s attitudes*** | ***Community’s attitudes*** | ***Religious leaders’ attitudes*** | ***Healthcare providers’ attitudes*** |
| --- | --- | --- | --- | --- | --- | --- | --- | --- | --- | --- |
|  |  |  | *Difference* | *Difference* | *Difference* | *Difference* | *Difference* | *Difference* | *Difference* | *Difference* |
| 1.Risk perception | .117 | 17.13% |  | .038 | .014 | .022 | .026 | .030 | .024 | .052* |
| 2.Effectiveness | .080 | 11.73% |  |  | -.024 | -.016 | -.011 | -.007 | -.014 | .014 |
| 3.Ease of Vaccination | .102 | 15.01% |  |  |  | .008 | .012 | .016 | .009 | .038* |
| 4.Own attitudes | .083 | 12.19% |  |  |  |  | .004 | .008 | .001 | .029* |
| 5.Family’s attitudes | .080 | 11.69% |  |  |  |  |  | .004 | -.003 | .025* |
| 6.Community’s attitudes | .076 | 11.13% |  |  |  |  |  |  | -.007 | .021* |
| 7.Religious leaders’ attitudes | .086 | 12.55% |  |  |  |  |  |  |  | .028* |
| 8.Healthcare providers’ attitudes | .058 | 8.57% |  |  |  |  |  |  |  |  |

*Note. (N* = 405), Diff. is computed by subtracting the raw importance weight of one predictor from the other.

*Indicates that the confidence interval for difference does not include 0, indicating that the difference of raw weights between the two predictors is significant.
